# Cauda Equina Syndrome Secondary to Seizure-Induced Spinal Fracture During Hyperbaric Oxygen Therapy: A Case Report

**DOI:** 10.5811/cpcem.63971

**Published:** 2026-10-13

**Authors:** Daniel Aloise, Joel Nash, Gregory Alfred, Rahib Iqbal, Eric Boccio

**Affiliations:** * Mount Sinai Medical Center, Department of Emergency Medicine, Miami Beach, Florida; † Nova Southeastern University, Dr. Kiran C. Patel College of Osteopathic Medicine, Davie, Florida; ‡ New York Medical College-School of Medicine, Department of Emergency Medicine, Valhalla, New York

**Keywords:** cauda equina syndrome, seizure-induced spinal fracture, hyperbaric oxygen therapy, beta thalassemia, case report

## Abstract

**Introduction:**

Hyperbaric oxygen therapy carries a risk of central nervous system oxygen toxicity, which can result in seizures. Seizure-induced spinal fractures are uncommon complications that occur during generalized convulsive activity. While often benign, lumbar vertebral body burst fractures may result in cauda equina syndrome. Additionally, patients with beta-thalassemia may have decreased bone mineral density due to ineffective erythropoiesis and bone marrow expansion increasing the risk for pathologic and traumatic fractures.

**Case Report:**

A 35-year-old man with beta-thalassemia major presented to the emergency department after a witnessed, first-time generalized tonic-clonic seizure during hyperbaric oxygen therapy for chronic venous ulcers. Initial evaluation revealed severe low back pain and midline lumbar tenderness. Computed tomography revealed an acute third lumbar vertebra (L3) burst fracture with retropulsion. The patient rapidly developed progressive right lower-extremity weakness and acute urinary retention. Magnetic resonance imaging revealed 70% loss of height of L3, severe thecal sac stenosis at L2–L3 and L3–L4, and a ventral dural hematoma resulting in stenosis at L4–L5. The patient was diagnosed with cauda equina syndrome and underwent emergent neurosurgical decompression.

**Conclusion:**

This case represents a rare convergence of multiple complications: central nervous system oxygen toxicity during hyperbaric oxygen therapy, seizure-induced lumbar burst fracture with retropulsion, and traumatic cauda equina syndrome in a patient with beta thalassemia. Physicians must maintain a high index of suspicion for vertebral fractures and monitor for evolving neurological deficits when managing the patient with acute back pain following seizure activity.

## INTRODUCTION

Seizure-induced spinal fractures represent an uncommon but well-documented complication of generalized convulsive activity. The violent muscle contractions generated during tonic-clonic seizures can produce sufficient axial force to cause compression or burst fractures, most commonly affecting the thoracolumbar junction.^1^ While most of these are minor compression fractures, severe complications including spinal cord compression or cauda equina syndrome can occur.^1^ Hyperbaric oxygen therapy, while an established treatment for chronic wounds, carries a risk of central nervous system (CNS) oxygen toxicity secondary to reactive oxygen species in brain tissue and decreased gamma-aminobutyric acid (GABA) concentrations.^2^,^3^ The risk of fracture is significantly compounded in patients with beta-thalassemia, who often suffer from multifactorial bone disease. Ineffective erythropoiesis causes bone marrow expansion, leading to cortical thinning and reduced trabecular bone tissue.^4^

This case presents a unique intersection of multiple rare complications involving a patient with beta-thalassemia major who sustained a third lumbar vertebra (L3) burst fracture and subsequent cauda equina syndrome following a seizure while undergoing hyperbaric oxygen therapy. Because clinical features may develop gradually and overlap with other pathologies, prompt recognition and emergent imaging are vital to prevent permanently disabling neurological sequela.

## CASE REPORT

A 35-year-old man with a history of chronic venous ulcers, beta-thalassemia major, and ureterolithiasis presented to the emergency department following a witnessed first-time seizure during hyperbaric oxygen therapy. The seizure was generalized tonic-clonic in nature, lasted less than one minute, and resolved spontaneously. There was no history of benzodiazepine or alcohol use, head trauma, known seizure disorder, or previous seizure during hyperbaric oxygen therapy.

On initial evaluation, vital signs were within normal limits. The patient was alert and oriented following the postictal period and only complained of low back pain. Physical examination revealed bilateral tongue lacerations. The head was normocephalic and atraumatic. Extraocular movements were intact, and pupils were equal, round, and reactive to light. The focused cardiac and pulmonary examinations were unremarkable. There was no midline cervical or thoracic spine tenderness. The patient complained of midline lumbar spine tenderness with right paralumbar tenderness. There were no palpable step-offs or muscle spasm. Motor strength was 5/5, sensation was intact in the bilateral lower extremities, and the patient had full range of motion while flexing and extending at the hips and knees bilaterally. There was an approximate 3 × 3-cm venous ulcer over the medial aspect of the right calf of the lower extremity. Given that the seizure occurred during hyperbaric oxygen therapy, oxygen toxicity was considered as a potential etiology. Computed tomography (CT) of the brain without contrast and CT of the abdomen and pelvis without contrast revealed an acute L3 vertebral body burst fracture (Image 1).

Incidentally, well-defined bilateral lobulated soft-tissue masses along the paravertebral regions of the spine consistent with extramedullary hematopoiesis were also observed on the CT abdomen and pelvis series (Image 2).

On reassessment following CT, the patient complained of worsening back pain. Physical examination revealed 4/5 motor strength with right hip flexion and knee extension. The patient was reporting suprapubic abdominal pain, and point-of-care ultrasound revealed a distended bladder, which was concerning for urinary retention. A Foley catheter was placed and emergent magnetic resonance imaging (MRI) of the lumbar spine without contrast was performed. The MRI redemonstrated the L3 vertebral body burst fracture with 70% loss of height and osseous retropulsion resulting in radiographic cauda equina compression (Image 3).

*CPC-EM Capsule*

What do we already know about this clinical entity?

*Hyperbaric oxygen therapy (HBOT) rarely causes oxygen toxicity seizures; however, seizure-induced vertebral fractures may occur.*

What makes this presentation of disease reportable?

*This rare case of an HBOT-related oxygen toxicity seizure resulted in a third lumbar vertebra burst fracture and cauda equina syndrome in a patient with beta-thalassemia.*

What is the major learning point?

*New back pain after a seizure warrants evaluation for vertebral fracture and evolving cauda equina syndrome, even without external trauma.*

How might this improve emergency medicine practice?

*Recognizing seizure-related spinal injuries early can expedite imaging, neurosurgical consultation, and decompression before permanent neurologic deficits develop.*

After neurosurgery was consulted, the patient was taken to the operating room for emergent L1–L5 posterior fixation and fusion, L2–L5 laminectomy, and L3 corpectomy with strut reconstruction. On hospital day 14, he developed worsening mid-back pain. Follow-up CT of the thoracic spine demonstrated bilateral L1 pedicle fractures extending into the L1 vertebral body. The patient subsequently returned to the operating room and underwent posterior T-10 pelvis fixation and fusion with cement augmentation of pedicle screws, as well as bilateral sacroiliac fixation and fusion.

The patient was referred to a spinal cord injury rehabilitation unit and discharged to a skilled nursing facility on hospital day 34. At discharge, neurologic examination demonstrated 4/5 strength in right ankle dorsiflexion and plantarflexion, 3/5 strength in right hip and knee flexion, and 2/5 strength in right knee extension. At six-month neurosurgical follow-up, strength had improved to 5/5 in right knee flexion and extension, ankle dorsiflexion, and plantarflexion, with 4/5 strength in right hip flexion. The patient denied saddle paresthesia and reported no bowel or bladder dysfunction.

## DISCUSSION

Hyperbaric oxygen therapy involves breathing 100% oxygen at pressures exceeding 1 atmosphere absolute (ATA), typically ranging from 1.8 to 3.0 ATA for therapeutic applications.^5^ While generally safe when administered according to standard protocols with treatment sessions limited to 120 minutes, hyperbaric oxygen therapy carries the risk of CNS oxygen toxicity, manifesting most dramatically as generalized tonic-clonic seizures.^6^ The incidence of oxygen toxicity seizures during hyperbaric oxygen therapy is relatively low. A recent retrospective cohort study of 634 sessions in patients with pre-existing seizure disorders reported only one seizure event.^7^ The pathophysiology of CNS oxygen toxicity is multifactorial. Recent evidence demonstrates that elevated partial pressure of oxygen increases the concentration of reactive oxygen species in neural tissues, which irreversibly alters cellular receptors and lowers the activity of glutamic acid decarboxylase, thereby reducing concentrations of the inhibitory neurotransmitter GABA.^6^ Additionally, elevated ambient pressure directly inhibits GABA and glycine receptors, contributing to rapid seizure onset.^6^

Central nervous system oxygen toxicity presents initially as a sequence of cardiorespiratory abnormalities including hyperoxic hyperventilation, which ultimately culminates in generalized seizures.^5^ The latent period before seizure onset is highly variable between individuals and within the same individual on different occasions, making prediction of toxic indications difficult.^5^ When seizures do occur during hyperbaric oxygen therapy, they are typically self-limited and cause no permanent neurological damage when the patient is immediately removed from the hyperbaric environment. However, the violent muscular contractions during these seizures can result in significant injury.

The mechanism of seizure-induced vertebral fractures involves violent forces generated by strong paraspinal muscles during tonic-clonic activity. A systematic review of seizure-related fractures found that thoracic and lumbar vertebral compression fractures accounted for 29% of all seizure-related fractures, second only to bilateral posterior shoulder fracture-dislocations (33%).^8^ However, a burst fracture—characterized by disruption of both anterior and posterior vertebral body columns with potential retropulsion of bone fragments into the spinal canal—may be a rare complication. A multicenter video-electroencephalogram study of 626 generalized convulsive seizures found vertebral body compression fractures in seven patients, yielding a risk of 1.1% per seizure (95% CI, 0.5–2.2%) and 1.7% per patient (95% CI, 0.8–3.3%).^9^ Importantly, these fractures occurred during the tonic phase of seizures and were accompanied by a characteristic audible cracking noise, with all affected patients reporting spontaneous back pain and increased pain on palpation of the affected spine.^9^

Several case reports have documented seizure-induced lumbar burst fractures. Youssef et al reported contiguous L1 and L2 burst fractures in a 35-year-old man following a witnessed tonic-clonic seizure while lying on a couch.^10^ McCullen and Brown described thoracic burst fractures with bilateral proximal humerus fractures secondary to seizure activity.^11^ Napier and Nolan reported a rare case of burst fracture resulting in conus medullaris syndrome following generalized motor seizures, emphasizing that despite the relative frequency of vertebral compression fractures, burst fractures with neurological sequelae are uncommon.^12^ Risk factors for seizure-induced fractures include the following: seizure severity and duration; use of antiseizure drugs known to decrease bone density; and family history of fractures.^8^ Patients with generalized tonic-clonic seizures have significantly higher injury rates compared to those with other seizure types. The presence of comorbidities, polytherapy drug regimens, and alcohol consumption have also been identified as independent predictors of seizure-related injuries.^13^

Cauda equina syndrome results from compression of the nerve roots in the lumbosacral spinal canal and presents with varying degrees of sensory loss, motor weakness, and bowel and bladder dysfunction. The classic clinical presentation includes urinary retention, saddle anesthesia of the perineum, bilateral lower extremity pain, numbness, and weakness. Decreased rectal tone may be a relatively late finding. The diagnosis of cauda equina syndrome can be challenging, as initial signs and symptoms are often subtle and may not present simultaneously. Cauda equina syndrome may be caused by spinal trauma resulting in injury at or below the level of L1. A study of 214 patients with traumatic cauda equina syndrome found that 52% were able to walk and more than 20% recovered bowel and bladder function by rehabilitation discharge, with median improvement in lower extremity motor score of four points.^14^ Shorter time from injury to rehabilitation admission was a significant predictor for improvement in both walking ability and bowel function.^14^ A study of 17 consecutive cases of cauda equina syndrome from low lumbar traumatic injuries found that 14 of 17 patients had satisfactory outcomes, with recovery of leg weakness occurring within four months and bladder and bowel function within three months.^15^ However, three patients with poor outcomes included two with gunshot wounds, highlighting the importance of injury mechanism.^15^ Time to surgery within 24 or 48 hours versus later did not represent a prognostic factor for recovery, and incomplete versus complete cauda equina syndrome did not show better recovery.^15^

Beta-thalassemia is associated with significant bone disease, representing a potential source of morbidity in this population. The pathophysiology is multifactorial, involving ineffective erythropoiesis causing bone marrow expansion with reduced trabecular bone tissue and cortical thinning, excessive iron loading causing endocrine dysfunction with increased bone turnover, and disease complications resulting in physical inactivity with subsequent reduction in optimal bone mineralization. Investigators have documented a high prevalence of osteoporosis in patients with transfusion-dependent beta-thalassemia.

## CONCLUSION

This case highlights the potential for severe neurological injury following a seizure induced by hyperbaric oxygen therapy. The patient’s convulsive episode, likely secondary to central nervous system oxygen toxicity, resulted in an L3 compression fracture and subsequent cauda equina syndrome. Although seizures induced by hyperbaric oxygen therapy are uncommon, clinicians should remain vigilant for this complication, particularly in patients with predisposing risk factors such as beta-thalassemia. As demonstrated in this case, a stepwise imaging strategy progressing from CT to emergent MRI is essential for identifying spinal cord or cauda equina compression. Fractures associated with neurological deficits require emergent surgical consultation, as timely decompression may remain an important determinant of functional recovery. In patients with underlying conditions such as beta-thalassemia, pretreatment assessment of bone health and careful monitoring for early signs of oxygen toxicity may help mitigate risk. Emergency physicians should maintain a high index of suspicion for vertebral injury after seizure activity, especially when accompanied by new back pain, focal spinal tenderness or deformity, or new neurologic deficits.

## Figures and Tables

**Image 1:**
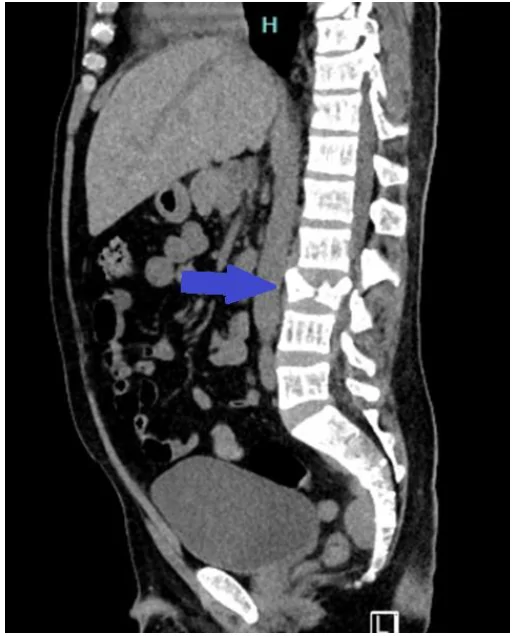
Computed tomography of the abdomen and pelvis showing an acute comminuted third lumbar burst fracture and severe spinal canal stenosis (arrow).

**Image 2:**
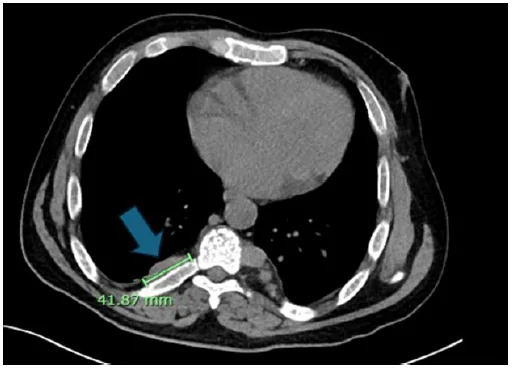
Computed tomography of the abdomen and pelvis showing multiple soft-tissue densities in the right parathoracic region, measuring 41.87 millimeters (mm) along its greatest axis (arrow, ruler).

**Image 3:**
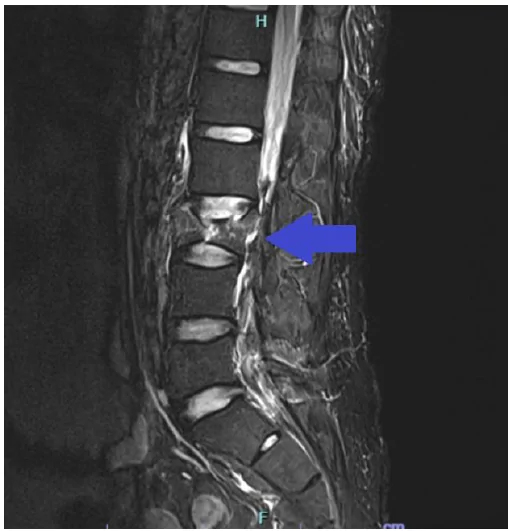
T2-weighted lumbar spine magnetic resonance image showing an acute third lumbar (L3) burst fracture with 70% loss of height, significant retropulsion, and severe thecal sac stenosis spanning L2–L5 secondary to a downstream ventral dural hematoma (arrow).
